# Preventive health checks in Australian general practice for women during mid‐life

**DOI:** 10.5694/mja2.52083

**Published:** 2023-08-24

**Authors:** Louise F Wilson, Annette J Dobson, Gita D Mishra, Jenny A Doust

**Affiliations:** ^1^ The University of Queensland Brisbane QLD

**Keywords:** Preventive health services, General practice, Chronic disease, Social determinants of health

The Australian mixed public and private health care model can lead to those most in need receiving the least or least effective care.^1^ We investigated whether this applied to preventive health checks in general practice for women in mid‐life. Two types of mid‐life preventive health checks are generally available in Australia: for people aged 40–49 years and at risk of type 2 diabetes or aged 45–49 years and at risk of chronic disease; and a heart health check (since 2019) (Supporting Information).

We analysed data for the Australian Longitudinal Study on Women's Health (ALSWH) cohort born during 1 January 1973 – 31 December 1978. Participants had been randomly selected from the Medicare database; women living in rural and remote areas were sampled at twice the rate of those in urban areas to facilitate comparisons.^2^ We analysed data derived from the most recent ALSWH survey completed by each woman before their 40th birthday (completed 2009, 2012, 2015, or 2018, depending on birth year) linked with Medicare Benefits Schedule (MBS) data for 1 January 2013 – 30 August 2021. We assessed associations between indicators of greater health care need and undergoing health checks in univariate and multivariable log‐binomial regression models (further details: Supporting Information). The ALSWH has ethics approval from the human research ethics committees of the University of Newcastle (H‐076‐0795) and the University of Queensland (2004000224).

Of the 14 247 women in the ALSWH 1973–1978 cohort, we excluded 93 who died before their 40th birthdays (0.6%), 746 who did not consent to MBS data linkage (5.2%), 2165 who responded only to the initial ALSWH survey in 1996 (at least fifteen years before their 40th birthdays; 15.2%), and 1081 for whom data were incomplete (7.6%). During 1 January 2013 – 30 August 2021, 1018 of the 10 162 included women (10%) had at least one health check; during 1 April 2019 – 30 August 2021, 44 women (fewer than 1% of those eligible) had heart health checks. In univariate analyses, women were more likely to have had health checks if they had risk factors for chronic disease (obesity, self‐rated fair or poor health), visited general practitioners four or more times a year, or did not have university degrees (Box). The associations were less marked after adjusting for socio‐demographic and health‐related factors (Supporting Information, table); after also adjusting for annual number of general practitioner visits, the major factors associated with health checks were not having a university degree and visiting general practitioners four or more times a year (Box). Of the 1018 women who had at least one preventive health check, thirteen (1.3%) had out‐of‐pocket expenses (ie, the fee exceeded the scheduled Medicare rebate for the visit).

Box 1 **The 1973–78 cohort of the Australian Longitudinal Study on Women's Health: univariate and multivariable regression analysis of associations with undergoing at least one preventive health assessment, 2013–21**

**Table:** 

Characteristic	No health assessment*	At least one health assessment*	Risk ratio (95% CI)	Adjusted risk ratio (95% CI)^†^
Number of women	9144	1018		
Age at survey (years), mean (SD)^‡^	36.3 (4.8)	36.1 (4.7)	—	—
Remoteness of residence^3^				
Major cities	5144 (67.4%)	591 (68.9%)	1	1
Inner regional	2518 (20.7%)	259 (18.9%)	0.9 (0.8–1.0)	0.8 (0.7–0.9)
Outer regional/rural/remote	1482 (11.9%)	168 (12.1%)	1.0 (0.8–1.2)	0.9 (0.7–1.0)
Highest education level				
Degree or higher	4469 (53.4%)	358 (39.2%)	1	1
Trade/diploma	2614 (26.7%)	343 (32.5%)	1.6 (1.4–1.8)	1.5 (1.3–1.7)
High school or less	2061 (19.9%)	317 (28.3%)	1.8 (1.6–2.1)	1.7 (1.5–2.0)
Language spoken at home				
English	8521 (91.3%)	954 (92.2%)	1	1
Language other than English	623 (8.7%)	64 (7.8%)	0.9 (0.7–1.2)	0.9 (0.7–1.2)
Marital status				
Married/de facto	6873 (74.8%)	744 (73.1%)	1	1
Separated/divorced/widowed	697 (7.5%)	102 (10.0%)	1.3 (1.1–1.6)	1.2 (1.0–1.4)
Never married	1574 (17.7%)	172 (16.9%)	1.0 (0.9–1.2)	0.9 (0.8–1.1)
Alcohol consumption^§^				
Never/rarely	3404 (36.2%)	430 (42.3%)	1.2 (1.0–1.3)	1.0 (0.9–1.2)
Low risk	5203 (58.0%)	544 (53.8%)	1	1
Risky/high risk	537 (5.8%)	44 (3.9%)	0.8 (0.6–1.1)	0.7 (0.5–1.0)
Smoking				
Non‐smoker	5290 (58.6%)	570 (57.4%)	1	1
Former smoker	2410 (26.2%)	264 (25.7%)	1.0 (0.9–1.2)	0.9 (0.8–1.1)
Current smoker	1444 (15.2%)	184 (16.9%)	1.2 (1.0–1.4)	1.0 (0.8–1.1)
Body mass index				
< 25 kg/m^2^	4609 (52.2%)	443 (44.9%)	1	1
25–29.9 kg/m^2^	2373 (25.6%)	268 (26.6%)	1.2 (1.0–1.3)	1.1 (0.9–1.3)
≥ 30 kg/m^2^	2162 (22.2%)	307 (28.5%)	1.4 (1.2–1.6)	1.2 (1.1–1.4)
Self‐rated health				
Excellent	1240 (14.0%)	109 (11.8%)	1	1
Very good/good	6927 (75.7%)	771 (74.8%)	1.2 (1.0–1.5)	1.1 (0.9–1.3)
Fair/poor	977 (10.3%)	138 (13.4%)	1.5 (1.2–1.9)	1.1 (0.9–1.4)
General practitioner visits/year^¶^				
Fewer than two	2104 (22.8%)	156 (14.3%)	1	1
2 or 3	2233 (24.9%)	214 (21.3%)	1.3 (1.0–1.5)	1.2 (1.0–1.5)
4 or 5	2586 (28.2%)	308 (30.2%)	1.5 (1.3–1.8)	1.5 (1.2–1.8)
6 or more	2221 (24.1%)	340 (34.1%)	1.9 (1.6–2.3)	1.7 (1.4–2.1)

CI = confidence interval; SD = standard deviation.

* Proportions weighted for area of residence to correct for oversampling in rural areas.

† Adjusted for all other variables.

‡ At the time of the most recent ALSWH survey completed before a woman's 40th birthday.

§ National Health and Medical Research Council guidelines: never/rarely drinker: less than one standard drink per moth drink/month; low risk: one drink per month to two drinks per day; risky/high risk drinker: three or more drinks per day.^4^

¶ Mean number in preceding two years, derived from linked Medicare Benefits Schedule data.

One limitation of our analysis is that the mean age of the women who completed the survey was 36 years (standard deviation, four years). However, a sensitivity analysis limited to the 7678 women aged 35 years or older at the time of the survey yielded similar results to our main analysis (data not shown).

Our findings contrast with Australian reports that fewer general practitioner services are provided to people with unhealthy behaviours,^5^ and that consultation times are generally shorter in areas of greater socio‐economic disadvantage.^6^ Our findings are consistent, however, with a study of health checks for Aboriginal and Torres Strait Islander people that found they were more frequent for people with the greatest medical needs and the highest levels of cardiovascular disease risk.^7^ The Medicare rebate for preventive health checks, at least for those at risk of diabetes and chronic disease, may be an adequate incentive for general practitioners to provide this care. Few women underwent heart health checks; the rebate for this service may be inadequate.

We found that general practitioners proactively provide preventive health care to those most in need, perhaps at least in part because of sufficiently high rebate levels.

## Open access

Open access publishing facilitated by the University of Queensland, as part of the Wiley – the University of Queensland agreement via the Council of Australian University Librarians.

## Competing interests

No relevant disclosures.

## Supporting information


Supplementary methods

## References

[1] Harris E , Harris MF . An exploration of the inverse care law and market forces in Australian primary health care. Aust J Prim Health 2023; 29: 137‐141. 36403292 10.1071/PY22160

[2] Dobson AJ , Hockey R , Brown WJ , et al. Cohort profile update: Australian Longitudinal Study on Women's Health. Int J Epidemiol 2015; 44: 1547. 26130741 10.1093/ije/dyv110

[3] Australian Bureau of Statistics . Australian Statistical Geography Standard (ASGS), volume 5: remoteness structure, July 2016 (1270.0.55.005). 16 Mar 2018. https://www.abs.gov.au/ausstats/abs@.nsf/mf/1270.0.55.005 (viewed July 2023).

[4] National Health and Medical Research Council . Australian alcohol guidelines: health risks and benefits. Oct 2001. http://web.archive.org/web/20060313170101/http://www.nhmrc.gov.au/publications/_files/ds9.pdf (viewed June 2023).

[5] Feng X , Girosi F , McRae IS . People with multiple unhealthy lifestyles are less likely to consult primary healthcare. BMC Fam Pract 2014; 15: 126. 24965672 10.1186/1471-2296-15-126 PMC4083035

[6] Furler JS , Harris E , Chondros P , et al. The inverse care law revisited: impact of disadvantaged location on accessing longer GP consultation times. Med J Aust 2002; 177: 80‐83. https://www.mja.com.au/journal/2002/177/2/inverse‐care‐law‐revisited‐impact‐disadvantaged‐location‐accessing‐longer‐gp 12098344 10.5694/j.1326-5377.2002.tb04673.x

[7] Butler DC , Agostino J , Paige E , et al. Aboriginal and Torres Strait Islander health checks: sociodemographic characteristics and cardiovascular risk factors. Public Health Res Pract 2022; 32: 31012103. 35290999 10.17061/phrp31012103

