# Supplementary material for: Preventive health checks in Australian general practice for women during mid‐life
Source: Med J Aust. 2023 Aug 24;219(9):429–31. doi: 10.5694/mja2.52083 (PMC10953379; doi:10.5694/mja2.52083)
Supplement: Supplementary file 1 — Supplementary methods [file MJA2-219-429-s001.pdf]

The Medical Journal of Australia

Supporting Information

Supplementary methods and results

This appendix was part of the submitted manuscript and has been peer reviewed.
It is posted as supplied by the authors.

Appendix to: Wilson LF, Dobson AJ, Mishra GD, Doust JA. Preventive health checks in Australian general practice for women during mid-life. *Med J Aust* 2023; doi: 10.5694/mja2.52083.

Supplementary methods

Study cohort

The Australian Longitudinal Study on Women's Health (ALSWH) is a prospective population-based cohort study that examines women's physical and mental health and their access to and use of health services. The study began in 1996 and included three cohorts of women, born in 1973–1978, 1946–1951 and 1921–1926. Participants for each cohort were randomly selected from the Medicare database; women living in rural and remote areas were sampled at twice the rate of women in urban areas to facilitate statistical comparisons.[1,2]

We analysed data for the 1973–1978 cohort. This cohort was first surveyed in 1996, and have been surveyed about every three years since (2000, 2003, 2006, 2009, 2012, 2015, 2018, 2021).

Medicare Benefits Schedule (MBS) Health assessments

We used linked MBS data to ascertain the first record of a health assessment for women after their 40th birthday (1 January 2013 – 30 August 2021; most recent MBS data available). Types of health assessments available to women during midlife are:

<table>
<tr><th>MBS item number [scheduled fee, Nov 2021] (consultation duration)</th><th>Type of health assessment</th><th>Frequency (MBS subsidy eligibility)</th></tr>
<tr><td rowspan="2">#224 [$49.40], #701 [$61.75] (≤ 30 min)
#225 [$114.80], #703 [$143.50] (> 30 to < 45 min)
#226 [$158.40], #705 [$198.00] (≥ 45 to < 60 min)
#227 [$223.75], #707 [$279.70] (≥ 60 min)</td><td>A type 2 diabetes risk evaluation for people aged 40-49 years (inclusive) with a high risk of developing type 2 diabetes as determined by the Australian Type 2 Diabetes Risk Assessment Tool</td><td>Once every three years to an eligible patient</td></tr>
<tr><td>A health assessment for people aged 45-49 years (inclusive) who are at risk of developing chronic disease</td><td>Once only to an eligible patient</td></tr>
<tr><td>#177 [$60.60], #699 [$75.75] (commenced April 2019, duration of ≥ 20 min)</td><td>Heart health assessment for a person who is 30 years or older (this age stipulation was added as an amendment in July 2021). Prior to this the age limits were 30 years and older for Aboriginal or Torres Strait Islander people and was 45 years or older for other Australians.</td><td>Once every 12 months (if other health assessments have not been claimed in that period)</td></tr>
</table>

Data collection

All variables, except for annual number of general practitioner visits, were assessed according to participants' survey responses. General practitioner visits were ascertained from linked MBS data (MBS item numbers: #3, #4, #23, #24, #36, #37, #44, #47). The number of visits in the two years preceding their 40th birthday was divided by 2 to estimate the mean annual number of visits.

Statistical analysis

Study baseline was the most recent survey women completed before their 40th birthday (as women only became eligible to receive a preventive health check at this age). The oldest women in the study turned 40 in 2013 (most recent survey: 2009), and the youngest at the end of 2019 (most recent survey: 2018). As not all women completed every survey, the time from the most recent survey to their 40th birthday varied; the mean period was four years (standard deviation, 4.4 years; range, 0–18

years), and 7678 women (76%) had completed a survey during the five years preceding their 40th birthday. We did not include women who responded only to Survey 1, as this would have meant a minimum of 15 years would have elapsed between the survey data and turning 40 years old.

Our analysis included data from 10,162 women from the 1973-78 cohort. Of the 14,247 women in the cohort, 93 were excluded because they died before their 40th birthday, 746 were excluded because they did not consent to linkage with MBS data, 2,165 were excluded because they only responded only to Survey 1 in 1996, and 1,081 were excluded because of missing data on one or more variables.

Baseline characteristics of women were compared by health assessment status. We used log-binomial regression to estimate risk ratios (RRs) with 95% confidence intervals (CIs) for the associations between socio-economic, health, and general practice factors and having a health assessment. First, we conducted univariate analyses for each variable. We then constructed three models: 1) a model that included the socio-demographic variables (remoteness of residence, highest education level, language spoken at home [English, other], marital status), 2) a model that included the socio-demographic variables of model 1, as well as alcohol consumption, smoking status, body mass index, and self-rated health, and 3) a fully adjusted model that included the variables of model 2, as well as mean annual number of general practitioner visits. The univariate analysis and model 3 are reported in the main article (Box); -models 1 and 2 are reported in this document (Table).

All analyses were undertaken in SAS for Windows 9.4.

References

1. Dobson AJ, Hockey R, Brown WJ, et al. Cohort profile update: Australian Longitudinal Study on Women's Health. *Int J Epidemiol* 2015; 44: 1547.

2. Lee C, Dobson AJ, Brown WJ, et al. Cohort profile: the Australian Longitudinal Study on Women's Health. *Int J Epidemiol* 2005; 34: 987-991.

Supplementary results

Table. The 1973–78 cohort of the Australian Longitudinal Study on Women's Health: multivariable regression analyses of associations with undergoing at least one preventive health assessment, 2013–21

	Adjusted risk ratio (95% confidence interval)	
**Characteristic**	**Model 1: Adjusted for socio-economic factors***	**Model 2: Adjusted for socio-economic and health factors†**
Remoteness of residence		
Major cities	1	1
Inner regional	0.8 (0.7–0.9)	0.8 (0.7–0.9)
Outer regional/rural/remote	0.9 (0.7–1.0)	0.8 (0.7–1.0)
Highest education level		
Degree or higher	1	1
Trade/diploma	1.6 (1.4–1.8)	1.5 (1.3–1.8)
High school or less	1.8 (1.6–2.1)	1.8 (1.5–2.1)
Language spoken at home		
English	1	1
Language other than English	0.9 (0.7–1.2)	0.9 (0.7–1.2)
Marital status		
Married/de facto	1	1
Separated/divorced/widowed	1.2 (1.0–1.5)	1.2 (1.0–1.5)
Never married	1.0 (0.8–1.1)	0.9 (0.8–1.1)
Alcohol consumption‡		
Never/rarely	–	1
Low risk	–	1.1 (0.9–1.2)
Risky/high risk	–	0.7 (0.5–1.0)
Smoking		
Non-smoker	–	1
Former smoker	–	0.9 (0.8–1.1)
Current smoker	–	1.0 (0.8–1.2)
Body mass index		
< 25 kg/m^2	–	1
25–29.9 kg/m^2	–	1.1 (0.9–1.3)
≥ 30 kg/m^2	–	1.3 (1.1–1.5)
Self-rated health		
Excellent	–	1
Very good/good	–	1.1 (0.9–1.4)
Fair/poor	–	1.2 (1.0–1.6)

* Adjusted for remoteness of residence, highest education level, language spoken at home and marital status

† Adjusted for remoteness of residence, highest education level, language spoken at home, marital status, alcohol consumption, smoking status, body mass index, self-rated health.
